# Induction and characterization of anti-tumor endothelium immunity elicited by ValloVax therapeutic cancer vaccine

**DOI:** 10.18632/oncotarget.15563

**Published:** 2017-02-21

**Authors:** Samuel C. Wagner, Thomas E. Ichim, Vladimir Bogin, Wei-Ping Min, Francisco Silva, Amit N. Patel, Santosh Kesari

**Affiliations:** ^1^ Batu Biologics, Inc. San Diego, CA, USA; ^2^ Department of Immunology, University of Western Ontario, London, Ontario, Canada; ^3^ Department of Surgery, University of Miami School of Medicine, Miami, FL, USA; ^4^ John Wayne Cancer Institute and Pacific Neuroscience Institute, Santa Monica, CA, USA

**Keywords:** angiogenesis, ValloVax

## Abstract

ValloVax is a placental endothelium derived vaccine which induces tissue-nonspecific antitumor immunity by blocking tumor angiogesis. To elucidate mechanisms of action, we showed that production of ValloVax, which involves treating placental endothelial cells with IFN-gamma, results in upregulation of HLA and costimulatory molecules. It was shown that in mixed lymphocyte reaction, ValloVax induces Type I cytokines and allo-proliferative responses. Plasma from ValloVax immunized mice was capable of killing *in vitro* tumor-like endothelium but not control endothelium. Using defined antigens associated with tumor endothelial cells, specific molecular entities were identified as being targeted by ValloVax induced antibodies. Binding of predominantly IgG antibodies to ValloVax cells was confirmed by flow cytometry. Further suggesting direct killing of tumor endothelial cells was expression of TUNEL positive cells, as well as, reduction in tumor oxygenation. Supporting a role for antibody mediated responses, cell depletion experiments suggested a predominant role of B cells in maintaining an intact anti-tumor endothelial response. Adoptive transfer experiments suggested that infusion of CD3+ T cells from immunized mice was sufficient to transfer tumor protection. Generation of memory T cells selective to tumor endothelial specific markers was observed. Functional confirmation of memory responses was observed in tumor rechallenge experiments. Furthermore, we observed that both PD-1 or CTLA-4 blockade augmented antitumor effects of ValloVax. These data suggest a T cell induced B cell mediated anti-tumor endothelial response and set the framework clinical trials through elucidation of mechanism of action.

## INTRODUCTION

Numerous approaches have been developed in attempts to selectively block tumor angiogenesis or induce collapse of tumor-associated blood vessels. While initial attempts such as development of endogenous inhibitors such as angiostatin and endostatin have failed, immunological means such as passive antibodies to VEGF (Avastin) have had success in terms of regulatory clearance and marketing approval. Drawbacks of Avastin include cardiotoxicity, development of resistance, has well as relatively poor survival advantage. Conceptually a more appealing method of inducing angiogenesis blockade would involve active immunization against several tumor endothelial associated antigens in the form of a polyvalent vaccine.

One major question that arises during attempts to induce active immunity to tumor associated endothelial is the “horror autotoxicus” potential of stimulating immunity towards non-malignant endothelium. We recently reviewed numerous works in which immunization to proliferating endothelial cells, whether syngeneic, allogeneic or xenogeneic results in selectivity of killing of tumors without damage to non-malignant tissues [1]. This is a fundamental point because numerous antigens found on tumor endothelial cells are also found on non-malignant cells, for example VEGFR is known to be associated with hematopoietic stem cell self-renewal. Despite this, as reviewed, immunization with VEGFR protein or plasmid does not result in ablation of hematopoietic stem cells, as would be expected. Accordingly multiple mechanisms must be at operation that discriminate tumor endothelial from cells expressing similar markers but are not under immunological attack and destruction as a result of the immunization. This is supported by clinical data in which immunization with HUVEC cells multiple times did not result in hematopoietic or other toxicities.

While numerous attempts have been made at immunizing tumor bearing mice to endothelial antigens, the mechanistic data behind development of immunity has not been elucidated. Wei et al, for example, demonstrated involvement of antibodies targeting alpha V beta III integrin in suppression of tumor angiogenesis following immunization of mice with xenogeneic HUVEC cells [2]. Other studies have implicated T cell responses [3–5]. Indeed, in some situations not only collaboration between T cells and B cells is required for successful antitumor immunity, but also epitope spreading is observed between initial immunity towards tumor endothelium, which is subsequently followed by immunity towards tumor antigens themselves. Thus there is a high degree of variability of biological mechanisms between different active immunotherapies which target the tumor vasculature.

ValloVax is a human placental endothelial cell derived product, which has demonstrated human safety in an initial pilot clinical trial [6], as well as being shown to effectively reduce tumor growth in lung cancer, melanoma, and breast cancer [7]. In contrast to other endothelium based vaccines, ValloVax has the unique properties of: a) Large donor supply. Since ValloVax is generated from placental endothelial cells, there exists a virtually unlimited supply of placentas, and additionally, each placenta is capable of generating a large number of doses; b) ValloVax is optimized for immunogenicity by pre-treatment with interferon gamma; and c) Placental endothelial is biologically naïve, thus allowing for a higher degree of plasticity. The enhanced plasticity allows for higher levels of surface marker manipulation subsequent to treatment with cytokines.

**Figure 1:**
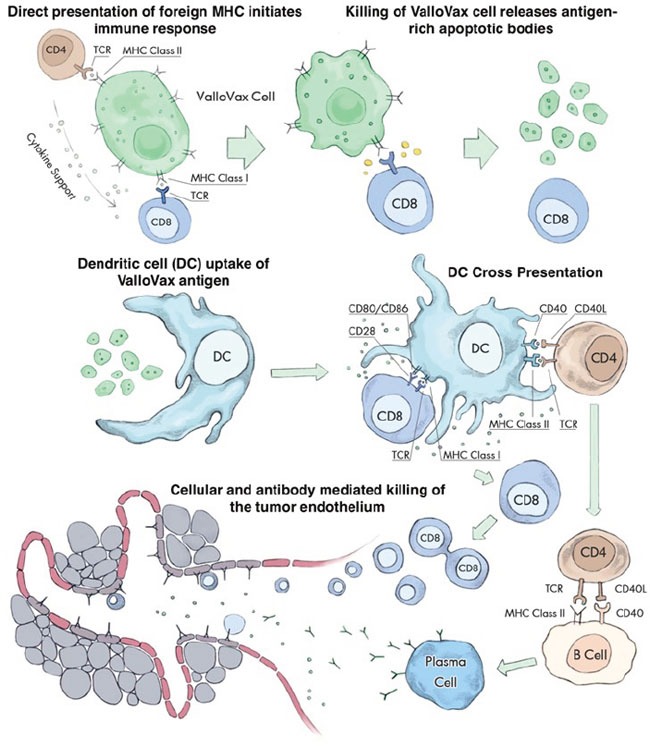
Multiple steps in the ValloVax-induced immune response Initial recognition of MHC expressed on the ValloVax cell induces antigen presentation via indirect recognition. Engulfment of apoptotic bodies released from the ValloVax cells in the process of phagocytosis. Cross presentation of ValloVax antigens, inducing an antigen-specific cellular and humoral immune response. The homologies between angiogenesis targets expressed on the ValloVax cell and tumor endothelium result in a cross reaction where the immune response spreads to induce killing of the tumor vasculature.

Initial experiments performed in this report sought to confirm whether interferon gamma treatment indeed increases immunogenicity of placental endothelial cells, which was assessed by quantification of Signal I molecules (HLA) and Signal II (CD40, CD80, and CD86). Subsequently function assessment was performed in terms of T cell allostimulatory activity as well as ability to elicit cytokines associated with tumor regression. The experiments subsequently assessed whether indeed selectivity of killing was occurring, specifically, whether *in vitro* generated tumor endothelium-like tissue was a target for antibodies generated *in vivo* from ValloVax immunized mice, and whether *in vivo* killing of endothelial cells was occurring. At a physiological level the assessment of tumor oxygenation was performed. From an immunological perspective, the dependence of ValloVax induced tumor regression on T cells or B cells was assessed through depletion and adoptive transfer studies. Furthermore, ability to synergize with clinically-used checkpoint inhibitors was performed. The current series of studies sought to establish a mechanistic basis for ValloVax efficacy, which will serve as the foundation for future clinical development. Based on previous immunological experiments describing mechanisms of allogeneic tumor vaccines, the following conceptual framework was used for designing the experiments (Figure 1)

## RESULTS

### Antitumor activity of ValloVax across histologically-distinct tumors

**Figure 2:**
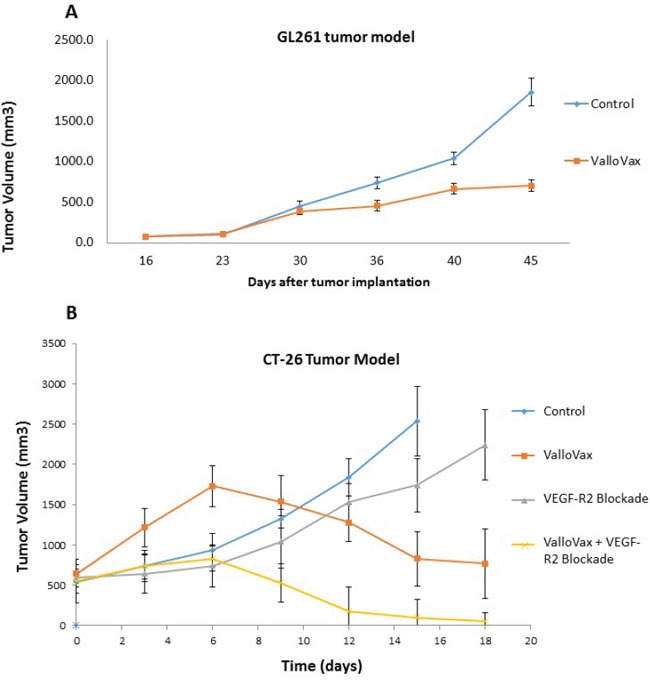
Efficacy of ValloVax across histologically distinct tumors GL-261 **A**. or CT-26 **B**. tumor cells were inoculated at time of vaccination or 10 days prior to vaccination, respectively, at a concentration of 1.7 × 10(6) or 5 × 10(5) cells per mouse. For GL-261 experiments, ValloVax was administered weekly, whereas for CT-26 experiments, vaccination was administered at day 10 post tumor inoculation and day 17. Tumor growth was assessed at the indicated timepoints.

In line with the hypothesis that ValloVax induces immunity to tumor endothelium, studies were conducted to determine whether administration of the vaccine would induce antitumor responses across histological tumor types. Previously we demonstrated that ValloVax vaccination inhibits growth of lung cancer, melanoma, and breast cancer [7]. In this study we utilized the GL261 glioma model and demonstrated suppression of tumor growth (Figure 2A). Additionally, using the CT-26 colon cancer model we demonstrated regression of established tumor (Figure 2B). In this model, superior activity of ValloVax was observed as compared to inhibition of VEGFR2 inhibition, suggesting the possible potency of active immunization towards a plurality of tumor endothelium associated antigens as compared to passive antibody transfer against one tumor vasculature associated antigen. These data support the possibility that ValloVax acts either by immunizing against antigens shared by all tumors, or by targeting a process common to all tumors, such as tumor angiogenesis.

### Interferon gamma pretreatment stimulates HLA and costimulatory molecule upregulation

Interferon gamma has previously demonstrated to induce upregulation of HLA I and HLA II on a variety of cell types [8–10]. In the creation of a cancer vaccine candidate the utility of allogeneic cells as immunogens has previously been reported in melanoma [11, 12], lung cancer [13, 14], renal cell cancer [15, 16], colon cancer [17, 18], and breast cancer [19–21]. It is known that correlations exist between HLA expression and immunogenicity of cancer cell vaccines [22]. Accordingly, while the rationale of pretreating cells with interferon is to upregulate immunogenicity, the actual determination of HLA upregulation, as well as effects on immunogenicity have not been reported. In a previous publication, antitumor efficacy of ValloVax treated with interferon gamma was demonstrated superior to untreated ValloVax [7], furthermore, increased expression of HLA I and II was demonstrated in endothelial cells pretreated with interferon gamma [23], but not in placental derived endothelial cells similar to ValloVax. As shown in Table 1, increased expression of HLA I and HLA II was observed in endothelial cells derived from all donor samples after treatment with interferon gamma for 48 hours. Interestingly upregulation of costimulatory molecules CD40, CD80, and CD86 was also observed.

**Table 1:** Upregulation of HLA and costimulatory molecules by IFN-gamma pretreatment

Flow Cytometric evaluation of markers upregulate by IFN-gamma treatment of EC						
Placenta Number	Treatment	HLA 1	HLA 2	CD40	CD80	CD86
**1**	Control	ND	+	+	+	ND
**1**	50 U	++	++	+++	++	+++
**1**	100 U	+++	++	+++	++	+++
**2**	Control	ND	ND	ND	+	ND
**2**	50 U	++	++	+++	++	+
**2**	100 U	+++	+++	+++	++	++
**3**	Control	ND	ND	ND	+	ND
**3**	50 U	++	++	+++	++	+
**3**	100 U	+++	+++	+++	++	++
**4**	Control	ND	ND	ND	+	ND
**4**	50 U	+++	+++	+++	+++	+
**4**	100 U	+++	+++	+++	+++	++
**5**	Control	ND	ND	ND	+	ND
**5**	50 U	++	+++	+++	++	+
**5**	100 U	+++	+++	+++	++	++

Placental endothelial cells were isolated from 5 different donors and treated for 48 hours with dilution buffer (control) 50 or 100 IU interferon gamma. Flow cytometry was utilized to assess expression of HLA I, HLA II, as well as costimulatory molecules CD40, CD80, and CD86. Quantification was performed by mean fluorescent intensity. ND = Not Detectable compare to isotype control; + = >25% MFI compared to isotype control; ++ = > 50% MFI compared to isotype control; +++ = > 75% MFI compared to isotype control; Experiments were done in triplicate in 3 different flow cytometry runs.

### Interferon gamma pretreatment of placental endothelial cells elicits hyperactive proliferative response from responding alloreactive T cells

**Figure 3:**
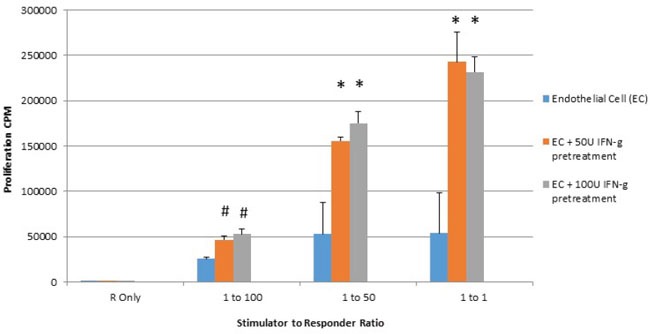
IFN-gamma pretreatment enhances proliferation of responding lymphocytes in mixed lymphocyte reaction (MLR) Placental endothelial cells were treated for 48 hours with dilution buffer (control) 50 or 100 IU interferon gamma and mitotically inactivated by irradiation. Cells were plated at the indicated stimulator:responder ratio in round bottom 96-well plates and cultured for 72 hours with tritiated thymidine added at the last 8 hours of culture. Proliferation was quantified by counts per minute. (*p < 0.01 as compared to control).

It is known that HLA molecules present on stimulator cell are triggers of alloreactive responder T cell proliferation in mixed lymphocyte reaction [24]. Additionally, costimulatory molecule expression on stimulator cells of the mixed lymphocyte reaction also are known to enhance alloreactive T cell proliferation [25, 26]. As seen in Figure 3, significant increases in T cell proliferation were seen at all stimulator to responder ratios subsequent to 48 hour treatment of placental endothelial stimulator cells to responders.

### Interferon gamma pretreatment of placental endothelial cells elicits Th1 cytokines from responding alloreactive T cells

**Figure 4:**
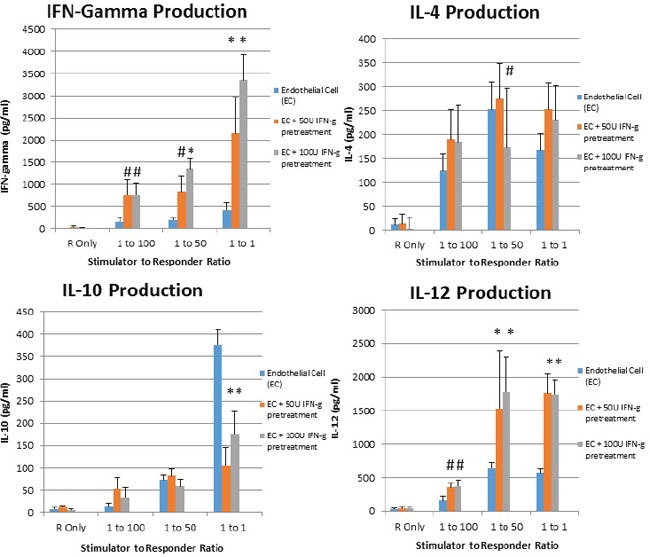
IFN-Gamma pretreated placental endothelial cells evoke Th1-Like response in MLR Placental endothelial cells were treated for 48 hours with dilution buffer (control) 50 or 100 IU interferon gamma and mitotically inactivated by irradiation. Cells were plated at the indicated stimulator:responder ratio in round bottom 96-well plates and cultured for 48 hours, after which production of the indicated cytokines was assessed by ELISA (*p < 0.01 as compared to control; #p < 0.05 as compared to control)

It is known that in mixed lymphocyte reaction, suppression of CD40, CD80, and CD86 is associated with reduction of T cell activation [27], stimulation of T regulatory cells [28], and upregulation of IL-10 [29]. Accordingly, given the upregulation of costimulatory molecules associated with interferon gamma pretreatment of placental endothelial cells, we sought to determine whether increased Th1 cytokines were produced when interferon gamma pretreated placental endothelial cells were utilized as stimulators. Interferon gamma pretreatment endowed placental endothelial cells ability to elicit upregulation of interferon gamma and IL-12 from responding T cells, as well as reduce production of IL-10. Interestingly, no modulation of IL-4 was observed (Figure 4).

### Selectivity of immune response induced by ValloVax is specific to tumor endothelium *in vitro*

Several reports exist from *in vitro* and *in vivo* studies, including clinical studies that immunity can be selectively induced against tumor endothelium (reviewed in [1]). In order to assess whether immunization with ValloVax can induce immunity towards tumor endothelium, an *in vitro* model of tumor endothelium was created. Previous studies have reported that culture of murine endothelial cells with a cocktail of TGF-beta, IL-10 and PGE-2 was sufficient to endow TEM-1 and Fas ligand expression on non-malignant endothelium [30]. In order to replicate a tumor endothelial environment we utilized several cocktails of conditioned media from tumor cell lines (LLC (lung cancer), B16 (melanoma), RM-1 (prostate cancer), GL-261 (glioma), 4T1 (breast cancer)), as well as tumor associated cytokines (PGE-2, TGF-beta, and IL-10). Bone marrow endothelial cells cultured with these cytokines and conditioned media were assessed for similarity to tumor endothelium by quantification of expression of Fas ligand, TEM-1, and ability to induce apoptosis in PHA activated splenocytes. Interestingly, the only combination to elicit reproducible upregulation of characteristics of tumor endothelial cells was 4T1 conditioned media combined with TGF-beta. Specific criteria for identifying whether the non-malignant endothelial cells actually resembled tumor endothelium included: a) Enhanced FasL expression (MFI >2000); b) Upregulated TEM-1 expression (MFI >2000); and c) 200% increased apoptosis of PHA and antiCD3 anti CD28 activated splenocytes compared to non-treated.

**Figure 5:**
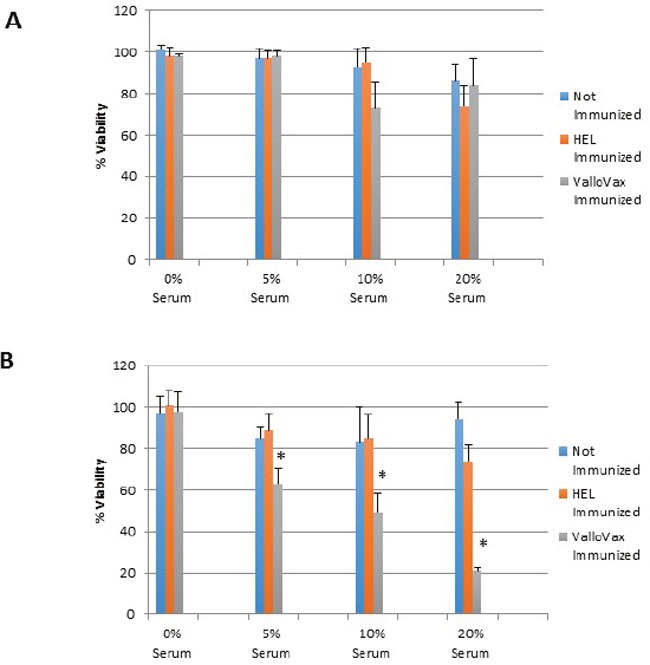
Selective killing of tumor endothelial cells by sera from ValloVax immunized mice Sera was collected from C57/BL6 mice that were either: unimmunized; immunized with HEL; or immunized with ValloVax. Sera was incubated with either healthy bone marrow derived endothelium **A**. or bone marrow derived tumor-like endothelium **B**. Viability was represented as percentage of absorbance with untreated cells. Six mice per group were utilized and *in vitro* culture was representative of triplicate experiments performed at least 3 times (*p < 0.01 as compared to control).

To assess whether sera from immunized mice displayed preferential cytotoxicity towards *in vitro* generated tumor endothelium, mice were immunized 4 times with 500,000 cells of ValloVax, or control immunization with hen egg white lysozyme (HEL) at 10 ug/mouse. Immunization occurred on days 0, 7, 14, and 21, with mice being sacrificed at day 30. Sera was collected from control unimmunized mice, ValloVax immunized mice, and control HEL immunized mice. As seen in Figure 5A, no toxicity of sera was detected against healthy endothelial cells, whereas toxicity was observed against tumor-like endothelial cells in Figure 5B. Interestingly, a dose-dependent toxicity was only observed with sera from immunized but not either of the control mice.

**Figure 6:**
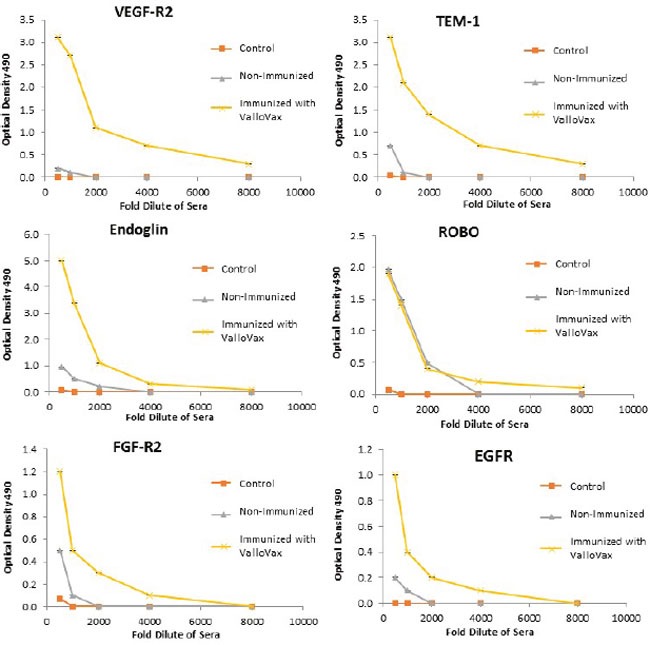
Antibody responses to tumor endothelial markers Sera from ValloVax immunized mice was used as a source of antibodies for quantification using ELISA detecting binding to indicated protein. Results are from sera of 6 mice per group and performed in triplicate.

**Figure 7:**
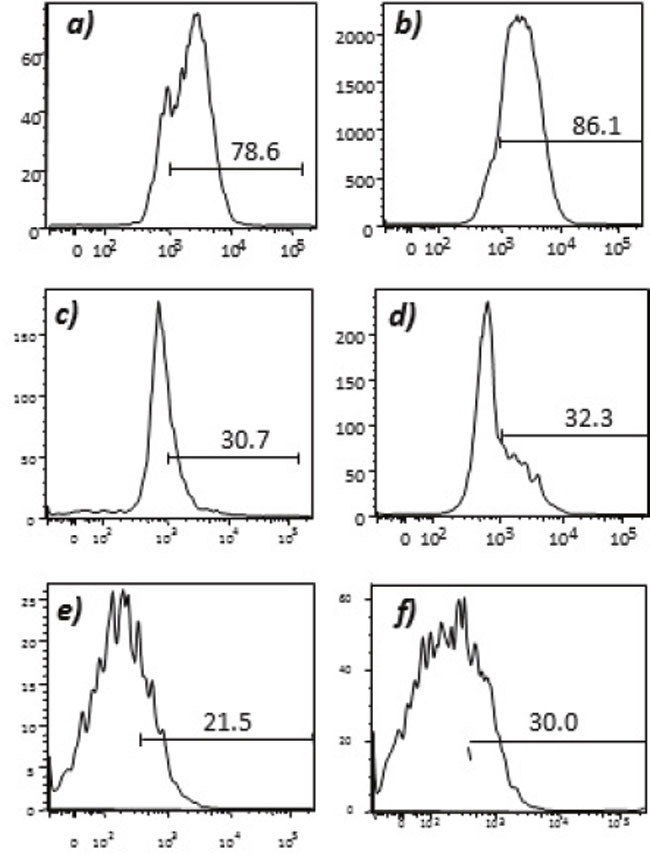
Expression of tumor endothelial markers on umbilical endothelial cells prior to interferon gamma treatment Purified CD31 endothelial cells were stained with; **A**. anti-VEGFR2; **B**. TEM-1; **C**. Endoglin; **D**. ROBO; **E**. FGF-R2; and **F**. EGF-R. Cells were gated on FSC and SSC and analyzed for the respective markers.

**Figure 8:**
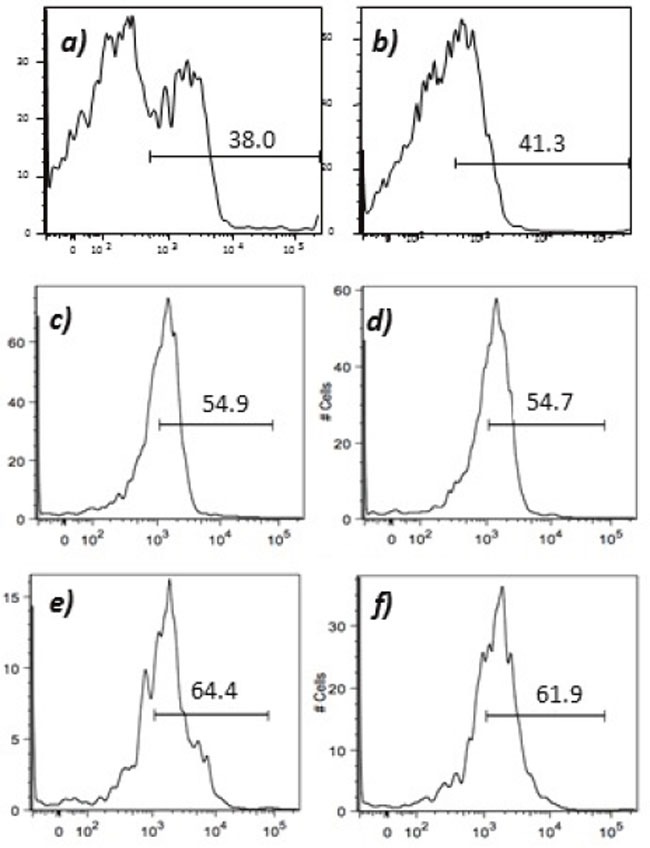
Expression of tumor endothelial markers on umbilical endothelial cells subsequent to interferon gamma treatment Purified CD31 endothelial cells were cultured for 48 hours in the presence of 100 IU/ml of interferon gamma and subsequently stained with; **A**. anti-VEGFR2; **B**. TEM-1; **C**. Endoglin; **D**. ROBO; **E**. FGF-R2; and **F**. EGF-R. Cells were gated on FSC and SSC and analyzed for the respective markers.

**Figure 9:**
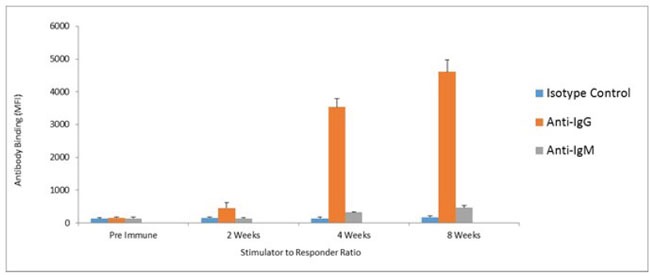
Assessment of class switching ValloVax cells where fixed with paraformaldehyde 0.5% and bound to 96 well ELISA plates. Plates where incubated with sera from immunized mice, and goat anti-mouse anti-IgG and goat anti-mouse IgM where used as secondary antibodies.

Humoral responses were assessed using an in house generated ELISA for antibody responses to VEGFR1, TEM1, CD105, ROBO, FGFR2 and EGF-R. Although varying degrees of antibody responses were seen, all animals demonstrated a significant induction of antibody generation as seen in Figure 6. We confirmed expression of these antigens on placental endothelial cells prior to treatment with interferon gamma (Figure 7), and subsequent to treatment (Figure 8). In order to identify whether antibodies generated in immunized mice actually bound to ValloVax cells, sera from immunized mice was collected at various timepoints post immunization and incubated with ValloVax cells. Characterization of antibody isotype binding was performed using anti-IgG and anti-IgM secondary antibodies. As seen in Figure 9, significant binding of IgG was noted, and antibody generation was time-dependent.

### *In vivo* killing of tumor endothelial cells

**Figure 10:**
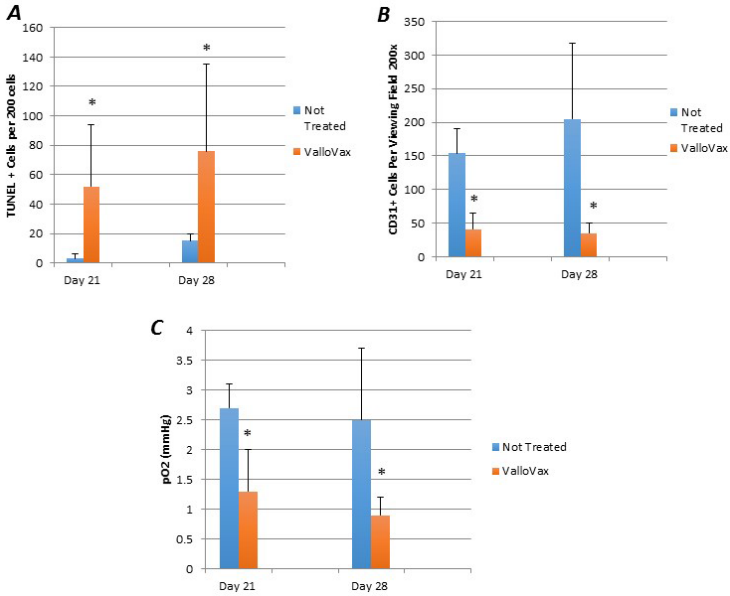
ValloVax induces reduction of tumor endothelial cells and reduces oxygenation Mice bearing LLC tumors were immunized with ValloVax and apoptosis was assessed by TUNEL staining **A**., Endothelial cell content was assessed by CD31 staining **B**., and tumor oxygen content was assessed by oximetry **C**. Values represent 12 mice per group (*p < 0.01 as compared to control).

Although previous studies have demonstrated inhibition and regression of tumor growth, mechanisms of tumor regression were investigated. Animals implanted with LLC tumors were immunized and sections where made to assess apoptosis with TUNEL staining. As seen in Figure 10A, an increased number of apoptotic cells were seen within the tumor tissue. Morphologically, apoptotic cells appeared to line the area adjacent to the blood supply of the tumor. Furthermore, quantification of tumor endothelial cells by CD31 staining revealed a reduced number of endothelial cells in the tumors, which correlated with tumor growth in Figure 10B. In order to definitively elucidate whether inhibition of tumor growth was occurring as a result of reduced oxygen supply, oximetry studies were conducted. Figure 10C illustrates reduction in oxygen content of ValloVax treated tumor bearing mice as compared to control.

### ValloVax mediated tumor regression requires B cells

**Figure 11:**
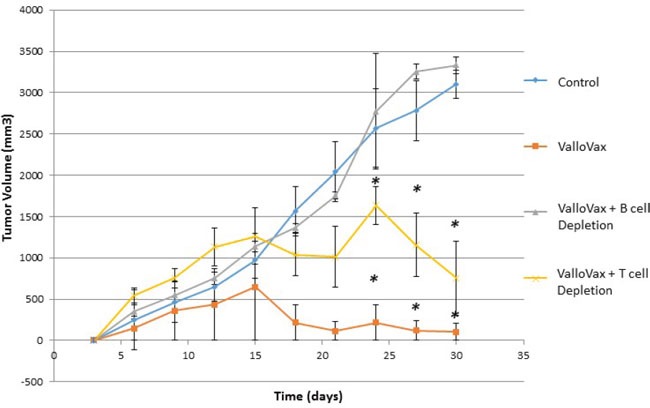
ValloVax mediated tumor regression is dependent on intact B cell compartment Mice where depleted of T cells or B cells by anti-CD3, and anti-CD20 depletion prior to and subsequent to LLC tumor administration. Mice were immunized with ValloVax and tumor regression was quantified. Numbers represent 12 mice per group (*p < 0.01 as compared to control).

Previous studies have demonstrated that antibodies may mediate anti-tumor endothelial responses mediated by immunization with endothelial cell antigens [31]. Accordingly, we assessed whether cytotoxicity to ValloVax cells *in vitro* was mediated by antibodies. To confirm whether effects were mediated by antibodies, T cells and B cells were depleted by antibody administration. As shown in Figure 11, tumor regression mediated by ValloVax was abrogated by B cell depletion but not T cell depletion, although a reduction of tumor inhibitory effects was observed in T cell depleted mice. These data suggest that there is some T cell contribution to the tumor inhibitory effect, which is in line with the classical immunological notion that B cells require T cell cytokine and costimulatory molecule support.

### T cells are required for adoptive transfer of tumor endothelial immunity

**Figure 12:**
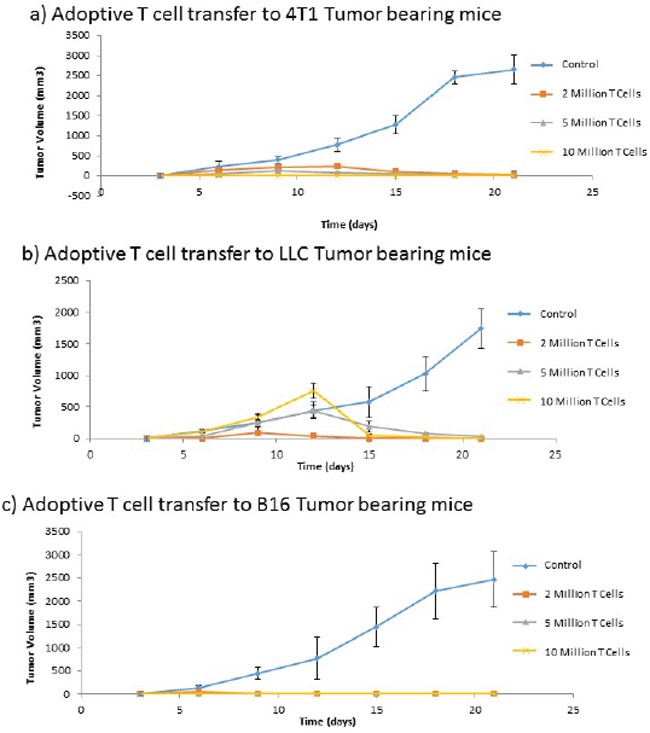
Adoptive transfer of immunity to ValloVax Purified T cells were extracted from immunized non-tumor bearing mice and transferred to naive mice that were subsequently inoculated with the designated tumor (**A**. 4T1, **B**. LLC, **C**. B16). Tumor regression was quantified. Numbers represent 12 mice per group.

Despite the need for antibodies in the induction of immune mediated tumor regression subsequent to ValloVax immunization, adoptive transfer experiments in 3 tumor models (B16, LLC, and 4T1) showed that CD3+ T cell transfer was capable of transferring immunity to all three tumors(Figure 12a–12c). This indicates that there may be a downstream activation of antigen-specific B cells subsequent to the adoptive transfer.

### Memory T cell responses to selected tumor endothelial antigens

**Figure 13:**
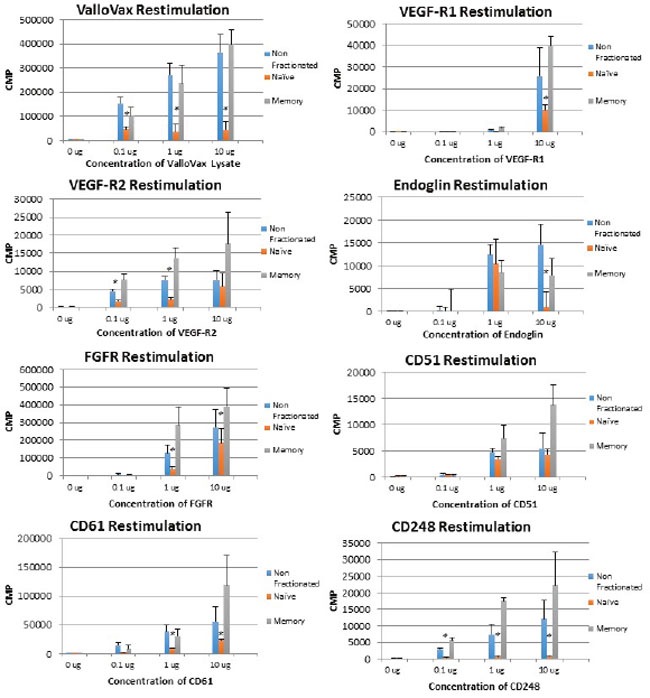
Memory T cell proliferation responses to tumor endothelial markers T cells were extracted from ValloVax immunized mice and separated into memory and naïve phenotype. Proliferation in response to indicated antigen is provided in CPM. Results are from lymphatic tissue of 6 mice per group and performed in triplicate (*p < 0.01 as compared to control).

**Figure 14:**
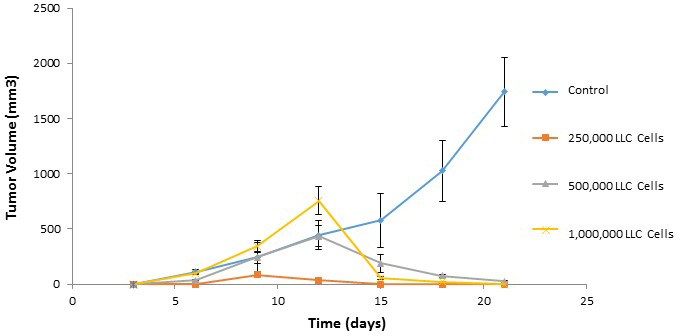
Adoptive transfer of immunity to naïve mice Immunized cells were collected from ValloVax treated mice as described in Materials and Methods. Recipient mice received 2, 5, and 10 million cells intravenously at time of tumor inoculation. For tumor rechallenge experiments, immunized mice that were tumor free on day 45-50 where challenged with 250,000, 500,000 or 1,000,000 tumor cells. Tumor growth was quantified.

In order to assess whether memory T cells were generated in response to ValloVax, and whether the recall proliferative response resided in the memory or the naïve compartment, lymph node and spleen cells were harvested and separated into CD44high cells, which represent memory compartment and CD44low cells which represent naïve T cells. As seen in Figure 13, with exception of CD105 restimulation, all recall responses where higher in the memory T cell compartment for the antigens tested, which were, ValloVax, VEGFR1, VEGR2, FGFR, CD105, CD51, CD61, and TEM 1. To conclusively demonstrate induction of immunological memory subsequent to adoptive transfer, mice were rechallenged with tumor cells after initial clearance of tumors, in all cases tumors did not reestablish (Figure 14).

### Stimulation of established tumor regression by combination of ValloVax with checkpoint inhibitors

**Figure 15:**
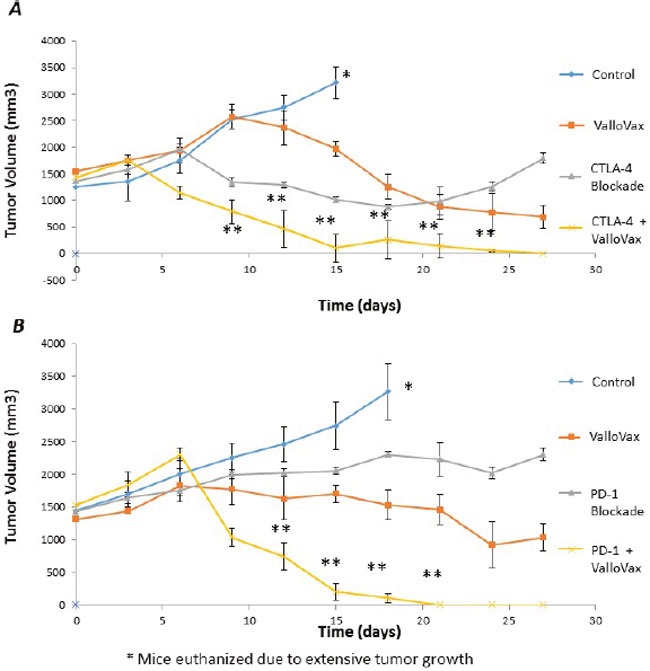
Enhancement of ValloVax activity by checkpoint inhibition Mice were implanted with LLC tumors, which were allowed to establish prior to immunization with ValloVax together with anti-CTLA4 **A**. or Anti-PD1 **B**. Tumor regression was assessed and each number indicates 12 animals per group. (*Animals were euthanized because of excessive tumor growth. ** p < 0.01 as compared to control, # p < .05 as compared to control).

Recent clinical trials have established the potency of checkpoint inhibitors against CTLA4 and PD1L in treatment of clinical tumors. Unfortunately checkpoint inhibitors possess the problem of lack of specificity to tumor tissue, thus in some cases causing autoimmunity. We sought to examine whether checkpoint inhibitors may potentiate ValloVax activity as assessed in an LLC model of established tumors. As seen in Figure 15, blockade of either CTLA4 or PD1 was not sufficient to induce regression of established tumors, whereas a synergy of effect was observed when combined with ValloVax.

## DISCUSSION

ValloVax is a GMP manufactured tumor endothelial-targeting immunotherapy candidate currently in clinical development by Batu Biologics for treatment of Non-Small Cell Lung Cancer (Investigational New Drug Application #16296). Previous studies have demonstrated antitumor efficacy of ValloVax in mouse models of breast and lung cancer, as well as melanoma. Confirmation of efficacy of ValloVax in tumors originating from histologically diverse tissues, particularly colon cancer and glioma, was performed in the current report. The reproducibility of this “universal vaccine” nature of ValloVax prompted us to evaluate mechanisms of ValloVax immunological activity, in part to identify cellular biomarkers that could be utilized in upcoming clinical trials, as well as to possibly explore means of augmentation of therapeutic effects. Although when compared to other tumor inhibitory immune vaccines [32–34], ValloVax appears more potent at reduction of tumor mass, translation of murine data to the clinical situation is usually characterized by loss of efficacy, and accordingly, we desired to possess possible means to augment efficacy during the clinical development of this candidate.

The concept behind generating ValloVax from placental endothelial cells was the involvement of these cells in not only creation of the vascular infrastructure supporting the maternal-fetal interphase, but also the unique immunological environment in the placenta, which resembles tumor vasculature from the perspective of both expressing FasL [35, 36], IL-10 [37, 38], TGF-beta [39], and other immune modulatory enzymes such as indolamine 2,3 deoxygenase [40]. Although other reports of endothelial cell vaccination have demonstrated immunity in humans without use of adjuvant, as quantified by antibodies to inoculation preparation [41], we sought to optimize immunogenicity of our endothelial vaccine by pretreatment with interferon gamma. In the current study found that placental endothelial cell treatment with interferon gamma increased immunogenicity of placental endothelial cells *in vitro*. Additionally, *in vitro* studies using artificially generated tumor endothelial lead to the finding of selective killing, which appeared to by IgG mediated, implying isotype and immunoglobulin class switching. The possibility of *in vivo* generation of antibodies suggests the feasibility of further epitope identification, as well as peptide vaccination.

We have identified several proteins that are found on ValloVax to which antibody responses are generated. These proteins comprised have previously been demonstrated to be present on tumor endothelium and have been used as targets of tumor endothelium vaccination: VEGFR2 [42–45], TEM-1 [46–48], Endoglin [49, 50], ROBO [51], FGF-R2 [5, 52], and EGF-R [53, 54]. The therapeutic induction of immune response toward the polyvalent mixture found in ValloVax suggests the possibility of synergy. Indeed, Miller et al noticed synergy between vaccination to EGF-R and VEGF-R2 at inhibiting tumor neovascularization [54]. Other have reported similar synergies in various animal models between different tumor endothelial specific antigens [55, 56]. In a previous publication we reported a pilot study of 3 solid tumor patients immunized with ValloVax. Antibody responses to VEGFR2, TEM-1, Endoglin, ROBO, FGF-R2 and EGF-R were observed. Most importantly, no elevations of toxicity associated enzymes where reported. This is of particular importance because of the theoretically possible concern of non-specific toxicity towards non-malignant endothelium or development of antigen-antibody complexes. Enzymes assessed included kidney enzymes, which were in normal ranges post immunization, which is reassuring, given that other immunotherapies which have off-target toxicity have been shown to elevate creatinine [57].

The concept of inducing immunity to a multi-valent or polyvalent immunization inoculum is superior from an immunological perspective to single antigen mediated vaccination from the perspective of tumor endothelial targeting given that recent evidence is suggesting not only tumor cells, but also tumor endothelium is capable of mutating. One study demonstrated that tumors with higher metastatic activity possess endothelial cells with higher mutating activity [58]. In some studies horizontal transfer of genetic material to endothelial cells has been demonstrated, thus causing variability of gene expression in the tumor vasculature [59].

*In vivo* studies demonstrated selective killing of tumor endothelial cells, through what appears to be apoptosis as demonstrated by TUNEL staining. Given that other forms of immunologically mediated cell death exist, such as autophagy [60, 61], the question is still open as to how such massive levels of tumor cell death are occurring although the TUNEL staining revealed relatively little death. One possibility is that the selective killing of endothelial cells results in regression of tumor through oxygen starvation, a possibility supported by our studies in which physiological levels of oxygen were severely reduced in tumor bearing mice.

We found that antibodies elicited to ValloVax cross reacted with major well known tumor endothelial cell antigens, thus demonstrating conclusively that potency and multivalent nature of the immune response generated. Supporting the concept of class switching during antibody development are the findings that memory T cells were obtained. Studies are underway to identify whether epitopes of the memory T cells are similar amino acid sequences to the B cell epitopes. The possibility remains that by linking T and B cell peptides together a synthetic vaccine can be generated that lacks the need for placental isolation and cell culture. One interesting observation was the transference of tumor immunity by the T cell compartment. This possibility indicates that tumor endothelial antigens are released at a basal rate from the tumor mass, which synergize with the adoptively transferred T cells. This suggests that processes already exist for immunological recognition of tumor endothelium, however T cell support is needed. Indeed other studies have shown the critical importance of T cells in supporting existing humoral tumor immune responses [62, 63]. Further support for immunological memory is the demonstration that mice which were rechallenged with tumor cells failed to develop tumors, even at concentrations of tumors as high as 10 million cells, which was observed in LLC, B16 and 4T1 systems.

Perhaps most striking were the tumor regression studies in which established tumors underwent considerable shrinkage subsequent to treatment with combination of checkpoint inhibitors and ValloVax. These data highly suggest the need to enter clinical trials with ValloVax alone, as well as in combination with classical checkpoint inhibitors. The possibility of enhancing specificity of checkpoint inhibitors while concurrently targeting the immune response to destroy tumor endothelium provides for enticing therapeutic possibilities. Another developmental possibility would be combination of ValloVax with CAR-T cell based approaches in order to augment efficacy in the treatment of solid tumors. The rationale would be that ValloVax induced anti-tumor endothelial responses would allow for enhanced intratumoral penetration of the CAR-T cells.

In conclusion, the current paper provides a mechanistic basis for utilization of ValloVax as a “second generation” Avastin in that an ongoing T cell response directed against proliferating tumor endothelium will lack the need for continued immunization, as well as possess ability to generate immunological memory and diversity, which should provide a significant therapeutic advantage to the current antiangiogenic treatments that are on the market now.

## MATERIALS AND METHODS

### Animals and cells

Female C57BL/6 and BALB/c mice aged 8–12 weeks were purchased from The Jackson Laboratory. Animals were housed under conventional conditions at the Animal Care Facility, University of Western Ontario, and were cared for in accordance with the guidelines established by the Canadian Council on Animal Care. A murine melanoma cell line established from a C57BL/6 mouse and designated B16F10 was obtained from the American Type Culture Collection (ATCC) and was maintained in RPMI 1640 medium (Sigma-Aldrich) with 10% FBS, l-glutamine, penicillin, and streptomycin at 37°C in 5% CO2. The murine mammary carcinoma 4 T1 cells (ATCC) were grown DMEM medium (Sigma-Aldrich) with 10% FBS, l-glutamine, penicillin, and streptomycin (complete DMEM) at 37°C in 5% CO2. Lewis Lung Carcinoma (LLC) is a murine lung carcinoma originating from C57/BL6 mice. The cells were maintained in RPMI 1640 supplemented with 10% fetal bovine serum, 2 mM glutamine (Gibco-BRL, Life Technologies, Inc.). The cell line was cultured at 37°C in a 5% incubator. GL261 cells murine glioblastoma cells (H-2^b^ haplotype) were obtained from ATCC and cultured in complete DMEM media. Cells were administered into C57/BL6 mice heterotopically into the subcutaneous tissue of the mouse flank at a concentration of 1.7 × 10(6) per inoculation. CT-26 murine colorectal cancer cells (ATCC) where grown in DMEM media and administered similarly as GL261 cells, with exception that inoculation was performed in BALB/c mice.

### Mixed lymphocyte reaction

Stimulator cells where placental endothelial cells that were treated for 48 hours with dilution buffer (control) 50 or 100 IU interferon gamma and mitotically inactivated by irradiation at 10 Gy. Cells were plated at the indicated stimulator:responder ratio, with 100,000 stimulators in round bottom 96-well plates and cultured for 72 hours with 1uC of tritiated thymidine added at the last 8 hours of culture. Proliferation was quantified by counts per minute.

For cytokine assessments, Quantikine ELISA kits (R & D Systems, Mannasses VA) were utilized. Conditioned media from MLR at 48 hours was used to assess cytokine production by responding cells.

### GMP preparation of vaccine

ValloVax was produced under Good Manufacturing Practices (GMP) at iBiologics. Full term human placentas were collected from delivery room under informed consent. Fetal membranes were manually peeled back and the villous tissue is isolated from the placental structure. Villous tissue was subsequently washed with cold saline to remove blood and scissors used to mechanically digest the tissue. Lots of 25 grams of minced tissue were incubated with approximately 50 ml of HBSS with proprietary enzymatic digestion mixture. The supernatant collected from all three incubations was then pooled and is poured through approximately four layers of sterile gauze and through one layer of 70 micrometer polyester mesh. The suspension was subsequently mixed with 10 ml of 90% Percoll to give a final density of 1.027 g/ml and centrifuged at 550 g for 10 minutes with the centrifuge brake off. The pellet was then washed in HBSS and cells incubated for 48 hours in complete DMEM media. After 3–4 passages cells were incubating in media containing 100 IU of IFN-gamma per ml. Subsequent to incubation cells were either used: a) unmanipulated; b) used as a lysate, with 10 freeze thaw cycles in liquid nitrogen, subsequent to which lysate was filtered through a 0.2 micron filter; c) mitotically inactivated by irradiation at 10 Gy; or d) inactivated by fixation in 0.5% formalin and subsequently washed.

### Immunization schedules and tumor assessment

For induction of tumor growth, 5 × 105 B16, LLC, or 4 T1 cells, American Type Culture Collection (Manassas, VA) cells were injected subcutaneously into the hind limb flank. Four weekly vaccinations of 5 × 105 test cells were administered subcutaneously on the contralateral side to which tumors were administered. Vaccination was performed on the day of tumor inoculation and on days 7, 14, and 21.

For GL261 studies, vaccination was performed on the day of tumor inoculation, followed by weekly administration. Vaccination dose was 1.7 × 10(6) cells per vaccination. For CT-26 studies, mice were immunized 10 days prior to tumor inoculation, as well as on days 10 and 17 subsequent to inoculation. Concentration of ValloVax cells was 5 × 10(5) cells per vaccination.

Tumor growth was assessed every 3 days by two measurements of perpendicular diameters by a caliper, and animals were sacrificed when tumors reached a size of 1 cm in any direction. Tumor volume was calculated by the following formula: (the shortest diameter2 × the longest diameter)/2.

### Adoptive transfer and tumor rechallenge

Donor mice syngeneic to recipient mice were immunized twice with ValloVax at days 0 and 7 at a concentration of 5 × 10(5) cells per injection. Mice were euthanized at day 14 by carbon dioxide asphyxiation followed by cervical dislocation. Lymph nodes where dissected mechanically and dissociated over a .45 sterile mesh in a solution of PBS with DNase 0.05 ug/ml. Cells were centrifuged twice in 5 ml of PBS and subsequently counted for viability. Recipient mice received 2, 5, and 10 million cells intravenously at time of tumor inoculation. For tumor rechallenge experiments, immunized mice that were tumor free on day 45-50 where challenged with 250,000, 500,000 or 1,000,000 tumor cells. Tumor growth was quantified.

### Memory cell assessment

C57/BL6 mice were immunized with Vallovax on days 1, 7, 14, and 21 and sacrificed on day 30. T cells were purified from single-cell suspensions of pooled lymphoid tissues (spleens and cervical, axillary, and inguinal lymph nodes). Naıve cells obtained by negative selection on CD4 T cell columns (R and D Systems), using the manufacturer's directions modified by adding anti-CD44 antibody to the antibodies supplied with the kit. Anti-CD44 antibodies selectively bind to CD44 hi memory activated cells and allow them to be retained in the column and removed from the suspension. Memory cells were purified by adding 5 mg/ml of sodium azide-free anti-CD62L antibodies (PharMingen) to those supplied with the CD4 column kit. CD62L is expressed selectively on naıve cells, and this marker was used to remove the CD62L naïve cells from the suspension. BM derived DC where added at a concentration of 100,000 cells per well from B6 mouse in 96 well plate, antigen (ValloVax lysate or protein) was added at concentrations of 0.1, 1, 10 ug. Furthermore memory (CD44+) or naïve cells (CD44-) T cells were placed at 100,000 cells per well. The mixture was incubated for 48 hours and proliferation was assessed by tritiated thymidine (1 microcurie/well) incorporation for 6 hours. Quantification of thymidine uptake was performed by measurement of counts per minute using scintillation counting (1205 Betaplate Liquid Scintillation Counter, WALLAC Inc., Gaithersburg, MD).

### Generation of *in vitro* tumor endothelium

BM CD31 selected cells were grown in Lonza EC media, and treated with TGF-beta (100 ng/ml) with 25% 4T1 supernatant. After culture for 48 hours, assessment of FasL and ability to induce apoptosis in PHA and anti-CD3/CD28 activated T cells was performed and compared to control BM CD31 selected cells, grown in Lonza EC media without TGF or 4T1 supernatant. These “tumor-like” EC where subsequently incubated for 48 hours with sera from immunized mice, 4 immunizations, sacrificed at day 30, used HEL immunization, same schedule as control. Assessed with ProMega Viability assay as percentage of control viability. Numbers are from 6 mice per group, *in vitro* done in triplicate x 3

### Immunohistochemistry

4-μm paraffin sections were deparaffinized in xylol and rehydrated in graded alcohol series. Endogenous peroxidase was inhibited using 3% H_2_O_2_ in methanol. The sections were then washed in distilled water and heated in a microwave oven (in citrate buffer 10 mM, pH 6, for CD31, and EDTA 1 mM, pH 7.5, for factor VIII) 15 min for epitope retrieval. No pretreatment was needed for D2-40. Slides were incubated first with normal horse serum (1/30 avidin 10%) for CD31 and with normal goat serum (10% avidin) for factor VIII and Fli-1 for 5 min and then in biotin for 10 min. Endogenous biotin was inhibited with a Vector Blocking kit (Vector Laboratories; Burlingame, CA). The slides were then incubated at 20C for 40 min with monoclonal antibodies for CD31, the slides were incubated overnight at 8C. The slides were incubated with anti-mouse/rabbit biotinylated bridging antibodies (dilution 1/200) for 30 min. Sections were then washed and incubated with standard avidin-biotin complex (ABC; DakoCytomation, Glostrup, Denmark) for 30 min. Antibody binding was revealed using H_2_O_2_ as a substrate and diaminobenzidine as chromogen. Counterstaining was performed with hematoxylin.

### Flow cytometry

Cells were suspended in phosphate buffered saline (PBS) and then incubated for 30 min at 4°C with the antibodies conjugated with FITC or PE against HLA I, HLA II, CD31, CD40, CD80, CD86, VEGFR-2, TEM-1, Endoglin, ROBO-4, FGFR-2, EGF-R and FasL. Flow cytometry analyses were performed using a FACSCalibur (BD Biosciences, San Jose, CA, USA). Acquired data were then analyzed by utilizing the Win-MDI software.

### ELISA for mouse immunizations and isotype analysis

Blood was extracted from the mice (~100 μl) by puncturing the radial venous with a 26G needle (Terumo Medical Corporation, Tokyo, Japan), and using heparinized micro-hematocrit tubes (Drummond Scientific Company, Broomall, PA, USA). The blood was centrifuged at 3,200 × g for 15 min to obtain the serum, which was stored at −20°C until analysis. For ELISA high-binding Nunc™ 96-well polystyrene plates (Thermo Fisher Scientific, Inc., Waltham, MA, USA), were coated overnight with 10 μg/ml of protein (VEGFR-1, TEM-1, CD105, ROBO-4, FGF-R2, EGF-R) at 4°C in carbonate/bicarbonate buffer (0.1 M; pH 9.6; Merck, Kenilworth, NJ, USA). Titrations where performed in PBS (0.1 M; pH 7.4) containing 2% bovine serum albumin (BSA; Sigma-Aldrich, St Louis, MO, USA). The plates were incubated for 3 h at 37°C. Subsequently, polyclonal rabbit anti-mouse immunoglobulin (Ig)G antibody conjugated to peroxidase was diluted to 1:10,000 in PBS (0.1 M; pH 7.4) and 2% BSA, was added to the plates. The antigen-antibody reaction was detected by addition of o-phenylenediamine (EMD Millipore) and a H_2_O_2_ substrate (EMD Millipore), which was dissolved in disodium phosphate (0.02 M, pH 5.0; EMD Millipore). The colorimetric reaction was read at 490 nm using a plate reader (Multiskan™ GO Microplate Spectrophotometer; Thermo Fisher Scientific, Inc.).

For assessment of antibody binding an isotype class switching, ValloVax cells where fixed with paraformaldehyde 0.5% and bound to 96 well ELISA plates. Plates where incubated with sera from immunized mice, and goat anti-mouse anti-IgG and goat anti-mouse IgM where used as secondary antibodies.
